# Lower attenuation and higher kurtosis of coronary artery calcification associated with vulnerable plaque – an agatston score propensity-matched CT radiomics study

**DOI:** 10.1186/s12872-023-03162-6

**Published:** 2023-03-27

**Authors:** Eric Po-Yu Huang, Huey-Shyan Lin, Yi-Chun Chen, Yi-He Li, Yi-Luan Huang, Yu-Jeng Ju, Hsien-Chung Yu, Gregory A. Kicska, Ming-Ting Wu

**Affiliations:** 1 Department of Radiology, Kaohsiung Veterans General Hospital, No. 386, Ta-Chung Dazhong 1st Road, Kaohsiung City, 813414 Taiwan; 2 Faculty of Medicine, School of Medicine, National Yang Ming Chiao Tung University, No.155, Sec.2, Linong Street, Taipei, 11221 Taiwan; 3 Department of Radiology, New Taipei City Hospital, No. 3, Sec. 1, New Taipei Blvd., Sanchong Dist., New Taipei City, 241204 Taiwan; 4 Department of Health-Business Administration, Fooyin University, 151 Chin-Hsueh Rd., Ta-Liao District, Kaohsiung, 831301 Taiwan; 5 Department of Psychology, National Taiwan University, No. 1, Sec. 4, Roosevelt Rd, Taipei, 10617 Taiwan; 6 Health Management Center, Kaohsiung Veterans General Hospital, No. 386, Ta- Chung 1st Road, Kaohsiung, 813414 Taiwan; 7 Section Chief of Thoracic Imaging, Radiology, University of Washington, 1959 NE Pacific Street, Seattle, WA 98195 United States of America; 8 Institute of Clinical Medicine, National Yang Ming Chiao Tung University, No.155, Sec.2, Linong Street, Taipei, 11221 Taiwan

**Keywords:** Acute Coronary Syndrome, Tomography, X-Ray computed, Coronary artery calcium, Coronary artery disease, Propensity score, Radiomics

## Abstract

**Background:**

Coronary artery calcification (CAC) burden assessed by Agatston score (AS) is currently recommended to stratify patients at risk for future acute coronary syndrome (ACS). Besides the CAC burden, the biostructure of CAC may also play a vital role in the vulnerability of CAC, which CT radiomics could reveal. Propensity-score matching of the traditional risk factors and CAC burden between the ACS and asymptomatic groups could radically remove biases and allow the exploration of characteristic features of CAC in ACS.

**Methods:**

We retrospectively identified 77 patients with ACS who had a CAC scan before percutaneous coronary intervention between 2016 and 2019. These 77 patients were one-to-two propensity-score matched for traditional risk factors of ACS and AS ranks to select 154 subjects from 2890 asymptomatic subjects. A validation cohort of 30 subjects was also enrolled. Radiomics features of each plaque were extracted and averaged in each person. Conditional logistic regression and area-under-curve analysis were used for statistical analysis.

**Results:**

A higher number of coronary segments involved, lower mean, median, first quartile, and standard deviation of attenuation, and increased kurtosis of attenuation of CAC were associated with the ACS group compared to the control group (*p* < 0.05 for all). Multivariable analysis showed that the lower median attenuation (OR = 0.969, p < 0.001) and higher Kurtosis (OR = 18.7, p < 0.001) were associated with the ACS group. The median attenuation and kurtosis significantly increase across AS ranks 1 to 4 (p = 0.001). The AUC of kurtosis (0.727) and median attenuation (0.66) were both significantly higher than that of the standard AS (AUC = 0.502) and the number of TRF (AUC = 0.537). The best cut-off of kurtosis at 2.74 yielded an accuracy of 74%, and the cut-off of median attenuation at 196 yielded an accuracy of 68%. The accuracy of kurtosis was 64%, and the accuracy of median attenuation was 55% in the validation cohort.

**Conclusion:**

After propensity-matching traditional risk factors and CAC burden, CT radiomics highlighted that lower median attenuation and higher kurtosis were the CAC characteristics of vulnerable plaques. These features improve the understanding of the biomechanics of CAC evolution and enhance the value of CAC scan in ACS risk assessment.

## Introduction

Coronary artery calcium (CAC) measurements on non-contrast ECG-gated CT are extensively used as a biomarker for coronary atherosclerosis burden and risk stratification of future coronary artery disease in preventive cardiology[1–6]. Agatston score (AS)[7] is the most widely used method for CAC quantification and is suitable for risk stratifications. The 2018 multi-society Guideline on the Management of Blood Cholesterol has given CT CAC testing for patients at risk of developing atherosclerotic cardiovascular disease a class IIa recommendation and suggested that it can guide treatment in preventive cardiology[8].

The noncalcified component of the coronary plaque is a significant limitation of the risk assessment of traditional CAC scans[9]. Coronary computed tomography angiography (CCTA) has proven better at identifying vulnerable plaques due to its ability to demonstrate noncalcified plaques [10–12], however, it requires the use of iodine-containing contrast at a high rate of injection. However, radiomics of CAC may bridge the gap by revealing additional textural features in vulnerable CAC plaques that are different from CAC in stable calcified plaque[13, 14]. While radiomics have been instrumental in improving CCTA’s ability to predict ACS[15, 16], there has only been one reported study using CT radiomics on pure CAC scan in a retrospective cohort, which found that radiomics score has mild incremental value compared to traditional AS in the prediction for major adverse cardiovascular events.[17].

We hypothesize that CT radiomics can help distinguish the CACs characteristics of vulnerable plaques on non-contrast CAC scans. Due to the less invasive nature of traditional CAC scans, they can be used on asymptomatic subjects. Since approximately 50% of all cardiovascular disease-related deaths have no prior cardiac symptoms or diagnoses[18], we conducted a propensity-matched study, matching the traditional risk factors (TRF) and CAC burden in terms of AS rank, to discover the CAC radiomics features that differ between ACS and asymptomatic groups. To date, this approach of matching the CAC burden to isolate the independent impact of CAC radiomics in vulnerable patients has not been reported.

## Subjects and methods

### Subjects and categories

**Fig. 1:**
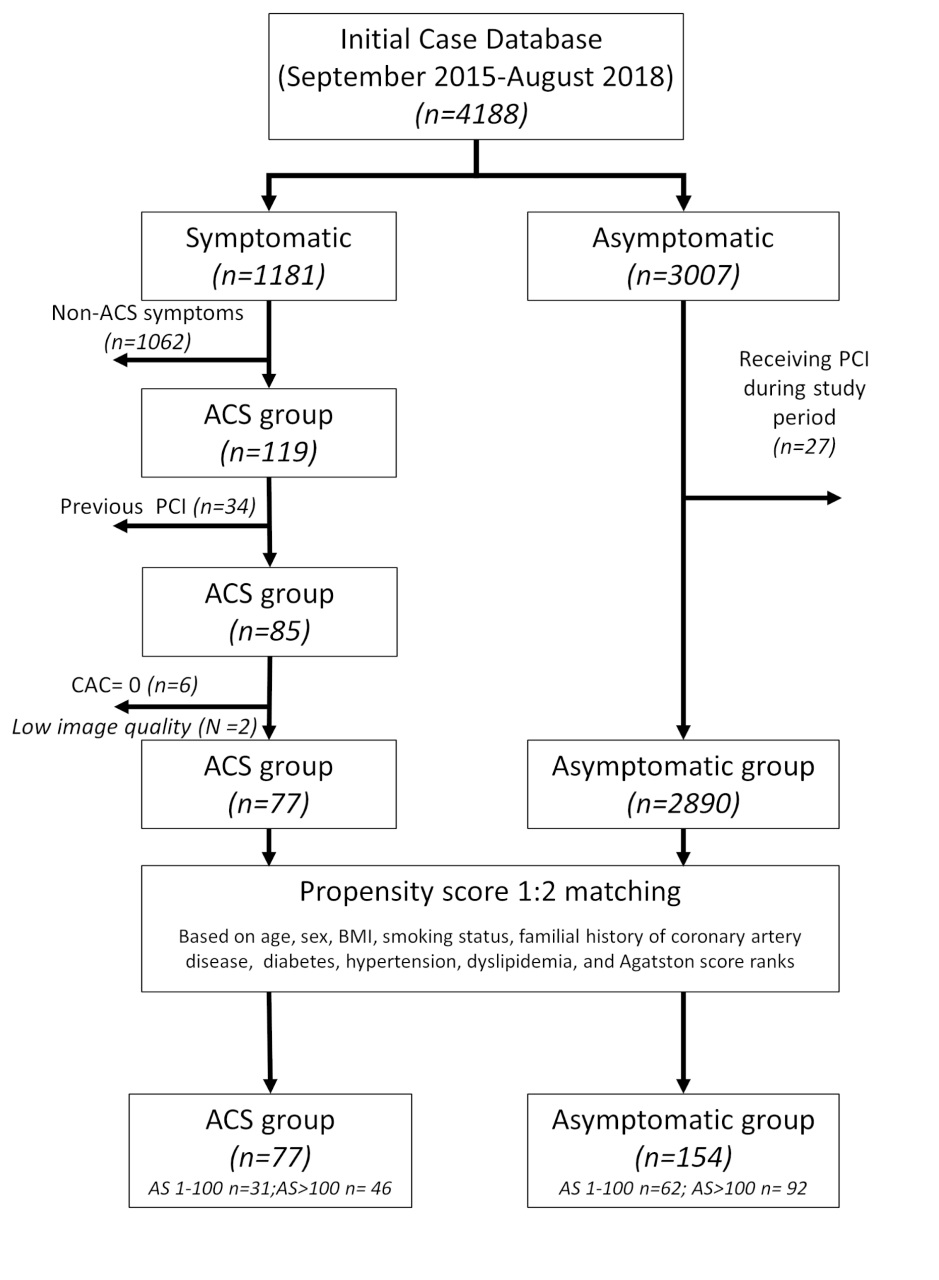
Flowchart of patient selection and propensity score matching process

In this retrospective propensity-matched study, the Radiological Information System of our institute was queried for cardiac CT exams between September 2015 to August 2018. The subject selection and propensity score matching process is summarized in Fig. 1. During this time, 4188 subjects receiving CAC CT scans were recorded. Among these, 1181 subjects were symptomatic, and 3007 were asymptomatic. Of the 3007 asymptomatic subjects who received a CAC scan as part of a voluntary cardiac healthcare program, 27 received elective percutaneous coronary intervention afterward and were excluded. From the 1181 symptomatic group, 119 patients ultimately developed ACS. Of the 119 patients, 34 had previously received the percutaneous coronary intervention and were excluded. Six patients with no CAC and 2 CT scans with poor image quality due to severe motion artifacts were excluded. 77 ACS patients and 2980 asymptomatic subjects were enrolled for the case-control propensity score matching. Parameters for propensity score matching included the traditional risk factors of ACS, i.e., age, sex, body mass index, smoking status, diabetes, hypertension, dyslipidemia, family history of ACS[8], and CAC burden using 3 AS ranks (1-100, 101–400, and > 400). The enrolled 77 ACS patients were 1:2 propensity score-matched with 154 asymptomatic subjects as the control group. A separate cohort of 10 ACS patients was enrolled later, and we performed a 1:2 propensity score match for 20 asymptomatic subjects to form the validation cohort.

### CT scan acquisition

The CT scans were performed on a 256-detector raw CT scanner (Revolution CT, GE Healthcare, Milwaukee, WIS, USA). We used the standard scanning protocols: tube voltage 120 kV, tube current 250–530 mA, automatically adjusted according to the preset noise level of 18, gantry rotation time 0.28 s, slice collimation 2.5 mm, and 250-mm field of view centered over the heart; the images were reconstructed at 75% of the R–R interval. The reconstruction was performed using a medium soft-tissue algorithm and 512 × 512 matrices with a 2.5 mm slice thickness.

### CAC analysis

Agatston score[7] was derived from CAC CT datasets using SmartScore 4.0 ™ (AW server 3.2, GE Healthcare). The entire coronary arterial tree was inspected and interrogated for the presence of calcified plaques by a CT technologist with 17 years of experience (CCC) under the supervision of a thoracic radiologist with 25 years of experience (MTW). A calcified plaque was defined as an area of 3 connected voxels with a CT attenuation ≥ 130 Hounsfield unit (HU) applying 3D connectivity criteria. Agatston score of each calcification was calculated[7], summed up, and converted into three ranks (1-100, 101–400, and > 400).

Radiomics analyses were performed using the LIFEx (version 6.1) package [19]. A semi-automated segmentation with the same ROI used for Agatston scoring was used to analyze radiomics parameters. Shape features, first-order histogram features, were extracted from the 77 1:2 propensity-matched group, which is composed of volume, mean, median, standard deviation, covariance, kurtosis, and skewness. Kurtosis is the peakedness of the pixel histogram, and a Gaussian distribution histogram has a kurtosis value of 3. Skewness is the measure of the asymmetry of a distribution, and a Gaussian distribution histogram has a skewness value of zero. [20].

### Statistical analysis

We used a generalized linear model to evaluate the effectiveness of our propensity match for univariate patient characteristics. Furthermore, effect size using Cohen’s d for continuous variables and matched odds ratio for categorical variables of each factor were also assessed. The values of the quantitative parameters of each plaque were then averaged for person-based analysis. Conditional logistic regression was used to determine univariate predictors for ACS. All variables entered into the single variable analysis were also added to the multivariate analysis, regardless of significance Forward conditional logistic regression was used to evaluate the relative importance of each factor found in this study. C-statistics of each independent variable were performed to determine cut-off values for prediction. A separate cohort of matched ACS and asymptomatic subjects was used to determine the predictive capabilities of the significant factors. The Delong algorithm was used to compare C-statistics between ROC curves. Discriminant analysis was performed by the lda() function of the MASS package in R (R core team, 2022) to find a linear combination beneficial for discriminating between the ACS group and the asymptomatic group. Statistical analysis was performed using SPSS 22 (IBM Corp. Released 2013. IBM SPSS Statistics for Windows, Version 22.0. Armonk, NY: IBM Corp.)

## Results

### Subject enrollment

A total of 231 subjects (77 ACS matched with 154 asymptomatic subjects) were enrolled; 207 men and 70 women, mean age of 61.5 years (range 31–80). Subject characteristics are shown in Table 1. All variables, including TRF and AS ranks, are well-matched between the groups. A small effect size is noted for all characteristics, suggesting low practical significance between the two groups. For the validation subjects (10 ACS matched with 20 asymptomatic subjects), 23 men and 7 women, mean age of 64.9 years (range 47–81). This study was approved by the Kaohsiung Veterans General Hospital’s Institutional Review Board. The Institutional Review Board waived the need for written informed consent from the participants.

**Table 1:** Clinical characteristics of 77 pairs 1:2 propensity matched subjects

	ACS (n = 77)	Asymptomatic (N = 154)	P value	Effect size
Age	61.6 ± 12.4	61.4 ± 11.9	0.18^a^	0.015^c^
Gender	69(89.6%)	138(89.6%)	1.00^b^	1.00 ^d^
Smoker	55(71.4%)	104(67.5%)	0.08^b^	1.17 ^d^
Diabetes Mellitus	24(31.2%)	49(31.8%)	0.84^b^	0.97 ^d^
Hypertension	50(64.9%)	96(62.3%)	0.44^b^	1.12 ^d^
Hypercholesterolemia	19(24.7%)	36(23.4%)	0.69^b^	1.07 ^d^
Agatston Score	173[34,575]	177[35,600]	0.59 ^a^	0.07 ^c^
Agatston Rank			N/A	N/A
1-100	31(40.3%)	62(40.3%)		
101–400	17(22.1%)	34(22.1%)		
> 400	29(37.7%)	58(37.7%)		

ACS: Acute coronary syndrome

Age visualized with Mean ± SD, Agatston score with Median [IQR]

^a^ Generalized linear model (linear) p-value;

^b^ Generalized linear model (logistic) p-value

^C^ Cohen’s *d*, values of 0.2, 0.5 and 0.8 for small, medium and large effect size

^D^ Matched OR, values of 1.5, 2.00 and 3.00 for small, medium and large effect size

### Texture analysis

From 71 radiomics features extracted, the key parameters were listed in Table 2, showing the comparison between the ACS and control groups. Single variable analysis indicates that the ACS group had a significantly higher number of segments involved, lower mean, median, first quartile, and standard deviation of attenuation, and increased kurtosis of attenuation compared to the control group. The multivariate analysis showed that the two independent factors of ACS were lower median and higher kurtosis of the attenuation.

**Table 2:** Plaque-based CAC features associated with acute coronary syndrome

	ACS		Control			Univariate				Multivariate		
Plaque-based (mean)	mean	SD	mean	SD		OR	95% CI	P-value		OR	95% CI	P-value
**AS > 0** N = 231 (77 groups)												
Plaque (N)	5.73	3.21	6.20	4.70		0.946	[0.857, 1.04]	0.269				
Segment (N)	4.74	2.42	4.27	2.44		1.235	[1.03, 1.49]	0.026*				
Vessel (N)	2.38	0.744	2.24	0.819		1.465	[0.928, 2.31]	0.101				
Plaque-based												
Volume (mL)	0.049	0.055	0.045	0.047		61.6	[0.026,1.44E5]	0.296				
Mean (HU)	222	40.1	245	53.7		0.981	[0.971, 0.991]	< 0.001*				
Median (HU)	203	28.9	223	41.3		0.970	[0.956, 0.984]	< 0.001*		0.969	[0.953, 0.986]	< 0.001*
First quartile (HU)	160	11.6	168	15.8		0.933	[0.902, 0.965]	< 0.001*				
SD	75.5	37.3	90.0	48.3		0.985	[0.975, 0.996]	0.005*				
COV	0.322	0.107	0.343	0.118		0.024	[0.001, 1.05]	0.053				
Kurtosis	2.80	0.581	2.36	0.483		17.3	[6.138, 48.7]	< 0.001*		18.7	[5.86, 60.0]	< 0.001*
Skewness	0.745	0.229	0.654	0.762		1.25	[0.804, 1.94]	0.321				

ACS: acute coronary syndrome, AS: Agatston score, CI: Confidence Interval, OR: Odds Ratio

*p < 0.05 indicates significance

Table 3 shows the median HU and Kurtosis across the 3 AS ranks and between the ACS and asymptomatic subjects. In both ACS and control subjects, median and kurtosis of attenuation significantly increased as AS ranks increased (p < 0.001 for all). Furthermore, median and kurtosis remained a significant differentiating factor between the groups regardless of AS ranks (p < 0.003 for all). Notably, the difference between the groups of median HU was most significant in the lower AS rank, while that of the kurtosis remained similar across the AS ranks.

**Table 3:** Comparison of median HU and kurtosis between ACS and control and between stratified AS ranks

**3 A Median HU**			p-value (ACS vs. control)
AS ranks	ACS	Control	
1-100 (N = 31、62)	179.41 ± 18.36	202.13 ± 41.19	< 0.001*
101–400 (N = 17、34)	203.87 ± 19.74	225.12 ± 33.15	0.003*
>401(N = 29、58)	226.78 ± 21.87	244.96 ± 34.17	0.001*
p-value (AS ranks)	< 0.001*	< 0.001*	
**3B Kurtosis**			p-value (ACS vs. control)
AS ranks	ACS	Control	
1–100 (N = 31、62)	2.49 ± 0.56	2.01 ± 0.31	< 0.001*
101–400 (N = 17、34)	2.74 ± 0.46	2.33 ± 0.34	< 0.001*
>401(N = 29、58)	3.18 ± 0.45	2.74 ± 0.42	< 0.001*
p-value (AS ranks)	< 0.001*	< 0.001*	

Comparison between ACS and control with paired generalized linear model (linear), and analysis of variance (ANOVA) between AS ranks

* p < 0.05 indicates significance

### Morphology

Due to the small size of some calcified plaque, not all the shape features were derived from every plaque. We tried several strategies and found the shape sphericity and compactness were insignificant regardless of different stratification (p > 0.05 for all).

### ROC curve analysis

**Fig. 2:**
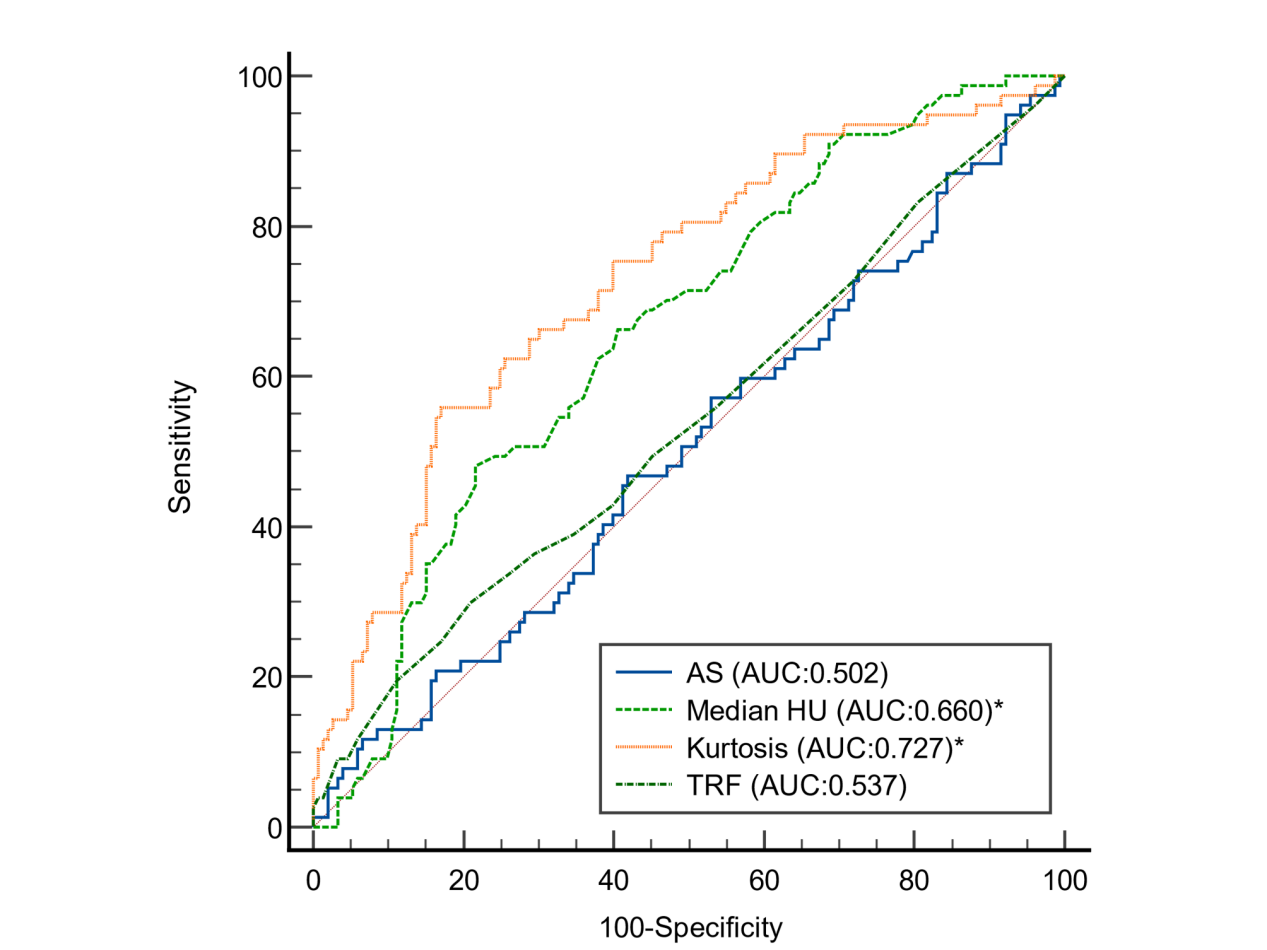
**Receiver operating characteristic curves of Agatston score, traditional risk factors, kurtosis of attenuation, and median of attenuation in the prediction of ACS in subjects with low CAC burden** Receiver operating characteristic (ROC) curves of Agatston score (AS), Agatston score, traditional risk factors, kurtosis of attenuation, and median of attenuation. Kurtosis (p < 0.001) and attenuation median were significantly superior to TRF and AS. There was no significant difference between kurtosis and median attenuation

C-statistics of kurtosis revealed the area under the curve (AUC) is 0.727 (95%CI 0.665–0.784). The best cut-off of kurtosis for ACS was 2.74, with an accuracy of 74.0%, sensitivity of 55.8%, and specificity of 83.12%. For the median HU, the AUC is 0.66 (95%CI 0.595–0.721), with the best cut-off of median HU set at 196, an accuracy of 68.0%, a sensitivity of 48.1%, and a specificity of 77.9%. The AUC of kurtosis and median HU are both significantly higher than that of the standard AS (AUC = 0.502), and the number of TRF (AUC = 0.537) (Kurtosis vs. AS and TRF p < 0.001, p < 0.001, respectively; median HU vs. AS and TRF p = 0.03, p = 0.04, respectively). (Fig. 2) Testing these cutoffs on a separate 1:2 matched cohort revealed that kurtosis has an accuracy of 64%, a sensitivity of 60%, and a specificity of 65%, whereas median HU has an accuracy of 55%, a sensitivity of 30%, and specificity of 55%. Combining kurtosis and median HU using a discriminant function, we found that using the following formula: (0.0217*median HU – 1.9059*Kurtosis), with a cutoff of 0 resulted in a sensitivity of 49.4% and a specificity of 90.9% with an accuracy of 77.1%.

## Discussion

This study has two unique features of study design that distinguish it from the previous CAC studies. First, we used propensity matching to eliminate the confounding effects of CAC burden and TRF to isolate the role of CAC radiomics associated with ACS. Propensity score matching is beneficial, especially in the field of cardiovascular research, to reduce systemic differences between cohorts without limitation in the number of covariates allowed[21]. This is also the first time CAC burden in terms of AS rank has been used as a factor in a propensity score-matched study. Second, plaque-based CAC features were derived by averaging each plaque’s features instead of summed-up person-based features. We found two independent and critical features of CAC in vulnerable plaques: lower median attenuation and higher kurtosis of CAC attenuation per plaque. To the best of our knowledge, this is the first report of pure CAC attenuation characteristics of vulnerable plaques isolated after matching the confounding impact of TRS and CAC burden.

We used propensity score match to select 154 out of 2890 asymptomatic subjects to (1) reduce the task burden of manually performing the radiomics analysis of all the 2890 asymptomatic subjects and (2) eliminate the confounding effect of AS ranks and TRF. The significant advantage of a propensity-matched study is that while the total number of patients enrolled in our study is small, the number of patients with the endpoint of ACS (N = 77 patients) is similar to that of the following extensive cohort studies. In the CONFIRM study, there were 58 mortality or non-fatal myocardial infarct cases from 3217 asymptomatic subjects[22]. In the Framingham heart study, there were 42 cases of coronary heart disease death or myocardial infarction from 1268 subjects with CAC > 0[3]. In the CAC radiomics of Framingham Heart Study, there were 30 events in the 318-discovery cohort and 29 events in the 306-validation cohort[17].

Previous studies have demonstrated that density scores that can be retrospectively derived from the Agatston score and volume score are independently associated with increased coronary heart disease [23, 24]. Our previous report also found that the CAC plaque in the ACS group had a lower and homogenous attenuation compared to subjects without symptoms or with chronic stable angina[25]. However, the impact of TRF and CAC burden among the three groups was not well eliminated. This study concluded that the lower median attenuation is an independent characteristic of the CAC of vulnerable plaques.

In subjects with similar AS, our ACS cohort’s lower median attenuation per calcified plaques can indicate decreased density compared to the higher-density plaques found in asymptomatic controls (Table 3 A). In both groups, the median HU increased as the AS ranks increased. These findings align with the current CAC plaque formation hypotheses that the early stage of plaque development has an increased risk of rupture, and is thus more vulnerable. After extensive and contiguous plaque calcification, plaques become less vulnerable and protected from rupture[26]. This notion is supported by a recent study using 18 F-NaF uptake on PET to identify that active calcification occurs predominantly at vessel locations with low-density calcification. These locations more often contain vulnerable plaque[27–29].

In addition, we found that the kurtosis of attenuation of the CAC plaque is new and independently associated with ACS. From Table 3B, the kurtosis was lowest in the asymptomatic group of ACS1-100 (2.01), representing the status of stable CAC formation. Kurtosis in the ACS group is significantly higher than in asymptomatic subjects regardless of AS ranks and increases with AS rank in both the ACS and asymptomatic cohorts. In other words, the greater the kurtosis, the more vulnerable the CAC plaque. We postulate that kurtosis reflects a stage in the evolution of CAC in coronary plaque that may be related to the vulnerability of the plaque.

In Fig. 2, the AUC of AS and TRF is close to 0.5, indicating that the study successfully removed the confounding effect of AS and TRF by propensity matching. In this scenario, a median CAC attenuation with a cut-off at 196 yielded a better AUC (AUC = 0.66, OR = 0.969) compared to AS and TRF, and kurtosis of attenuation with a cut-off value of 2.74 was able to deliver an even better AUC in all subjects (AUC = 0.727, OR = 18.7). The predictive ability of kurtosis alone is comparable to that of a composite radiomics-based score derived from a retrospective cohort study[17].

This study has several limitations. (1) Although we used propensity score matching, biases outside the scoring, such as precise medication and laboratory data, could not be eliminated. (2) Analysis of these CAC radiomics was manually completed using LIFEx software. As per the definition of CAC, plaques are at least 3 adjacent pixels with attenuation > 130, some plaques were insufficient in size and shape for 2nd -order texture analysis, and our radiomics analysis only contained the 1st -order texture parameters. (4) We did not include medication information in our study, nor were medications matched between our cohorts. As such, the effects of medication such as statins could not be evaluated in this study. (5) We did not evaluate the interval time between our CAC scan and the diagnosis of acute coronary syndrome in the patient group, and future Kaplan-Meier analysis may improve our understanding of the relationship between CAC features and ACS event, and (6) All participants were enrolled in a single medical center; our results may not generalize to other regions or ethnic groups. A future automatic AI program used in an external validation study with a larger population is planned to confirm these preliminary findings.

## Conclusion

In this propensity-matched study, we found that higher kurtosis and lower median attenuation per plaque were independently associated with the ACS group than asymptomatic subjects with matched TRF and CAC burdens. With this approach, the critically relevant CAC radiomics was isolated and has provided insight into the biomechanics of CAC formation in vulnerable plaques. These features may meet the emergent need to predict vulnerable plaques using current standard CAC scans.

## Data Availability

The data that support the findings of this study are available from the corresponding author, M.T.W. upon reasonable request.

## Abbreviations

ACS: Acute coronary syndrome
AS: Agatston score
CAC: Coronary artery calcium
HU: Hounsfield Units
PCI: Percutaneous coronary intervention
SD: Standard deviation
TRF: Traditional risk factors
